# Marine Rare Actinobacteria: Isolation, Characterization, and Strategies for Harnessing Bioactive Compounds

**DOI:** 10.3389/fmicb.2017.01106

**Published:** 2017-06-15

**Authors:** Dipesh Dhakal, Anaya Raj Pokhrel, Biplav Shrestha, Jae Kyung Sohng

**Affiliations:** ^1^Department of Life Science and Biochemical Engineering, Sun Moon UniversityAsan-si, South Korea; ^2^Department of BT-Convergent Pharmaceutical Engineering, Sun Moon University Asan-siSouth Korea

**Keywords:** marine rare actinobacteria, bacterial characterization, bioactive compounds, metagenomics, host engineering

## Abstract

Actinobacteria are prolific producers of thousands of biologically active natural compounds with diverse activities. More than half of these bioactive compounds have been isolated from members belonging to actinobacteria. Recently, rare actinobacteria existing at different environmental settings such as high altitudes, volcanic areas, and marine environment have attracted attention. It has been speculated that physiological or biochemical pressures under such harsh environmental conditions can lead to the production of diversified natural compounds. Hence, marine environment has been focused for the discovery of novel natural products with biological potency. Many novel and promising bioactive compounds with versatile medicinal, industrial, or agricultural uses have been isolated and characterized. The natural compounds cannot be directly used as drug or other purposes, so they are structurally modified and diversified to ameliorate their biological or chemical properties. Versatile synthetic biological tools, metabolic engineering techniques, and chemical synthesis platform can be used to assist such structural modification. This review summarizes the latest studies on marine rare actinobacteria and their natural products with focus on recent approaches for structural and functional diversification of such microbial chemicals for attaining better applications.

## Introduction

Actinobacteria are Gram-positive bacteria with high GC contents in DNA. They have characteristics presence of intracellular proteasomes, and spores if present are exospores (Cavalier-Smith, 2002). The order *Actinomycetales* under phylum *Actinobacteria* includes major producer strains of diverse bioactive compounds. *Actinomycetales* includes 11 suborders *viz. Actinomycineae, Actinopolysporineae, Catenulisporineae, Corynebacterineae, Glycomycineae, Jiangellineae, Micromonosporineae, Propionibacterineae, Pseudonocardineae, Streptomycineae*, and *Streptosporangineae* (http://www.bacterio.net/-classifphyla.html). The genus *Streptomyces* under sub-order *Streptomycineae* have been characterized as most important producer of bioactive microbial metabolites (Berdy, 2005). Recently, previously underexplored genera are reported as important resources of diverse bioactive metabolites (Tiwari and Gupta, 2013). These so called rare-actinobacteria are commonly categorized as strains other than *Streptomyces* (Berdy, 2005) or actinobacteria strains with less frequency of isolation under normal parameters (Lazzarini et al., 2001; Baltz, 2006).

The un-explored and under-explored habitats including marine ecosystems are believed to be rich sources of such rare actinobacteria, with tremendous potential to produce interestingly new compounds (Hong et al., 2009). These marine actinobacteria with potential of producing bioactive compounds have attracted major attention to search for unique compounds with pharmaceutical and biotechnological applications (Bull and Stach, 2007; Subramani and Aalbersberg, 2013; Azman et al., 2015). Recently, there are reports on the discovery of rare actinobacteria from wide range of terrestrial and aquatic locations, including deep seas (Goodfellow et al., 2012). Reports on the analysis of geographical origins of the marine rare actinobacteria, with special focus on the isolation of specific compounds, and precise bioactivities are predominant indications of increasing global interest on the natural compounds from marine rare actinobacteria (Blunt et al., 2007).

## Isolation and characterization of marine rare actinobacteria

Generally, for uncovering the marine rare actinobacteria, isolation efforts have been focused on rare locations as deep-sea sediments to obtain new marine diversities (Fenical and Jensen, 2006). The specialized sampling techniques using sophisticated equipment (Fenical and Jensen, 2006), remotely operated vehicles (Pathom-Aree et al., 2006) and even human (Bredholdt et al., 2007), have provided easy access to unprecedented microbial diversity. However, marine rare actinobacteria are usually difficult to culture compared to their terrestrial counterparts mostly due to their special growth requirements (Zotchev, 2012) or unknown culture conditions. It has been observed that hardly <2% of bacterial cells can form colonies by conventional plate cultivation. A large number of them belong to “viable but not culturable” (VBNC) strains (Bernard et al., 2000). Recently, strategies such as mimicking the natural environment in terms of pH, oxygen gradient, nutrient compositions, *etc* is employed. With these improvements, some previously VBNC species can now be grown with more efficiency (Kaeberlein et al., 2002; Zengler et al., 2002; Vartoukian et al., 2010; Stewart, 2012).

Moreover, the laborious microscopic techniques are being replaced with techniques utilizing recent advances in genomics, proteomics, and bioinformatics for identification and characterization of microbial diversity in robust manner (Rastogi and Sani, 2011). The genomic analysis by genetic fingerprinting (Nübel et al., 1999), DNA-DNA hybridization techniques (Pinhassi et al., 1997), and the construction of metagenomic library and sequencing (Kisand et al., 2012) have been employed for identifying and characterizing the diversity within marine samples. The development of next generation sequencing (NGS) (Webster et al., 2010) and nanopore sequencing (Deamer et al., 2016) has made the process robust and less time consuming. The analysis of RNA expression and regulation using metatranscriptomics (Ogura et al., 2011) or determination of protein profile by metaproteomics (Slattery et al., 2012) can be directly linked to available genome in the database. The coupled metagenomics and metatranscriptomic analysis was successfully used for determining the microbial communities in deep sea water of the North Pacific Ocean (Wu J. et al., 2013). Thus, the combination of both culture dependent (grow and isolate) and culture independent (analysis of nucleic acids and proteins) approaches have revolutionized the characterization and isolation of diverse marine organisms including rare actinobacteria (Hirayama et al., 2007; Zeng et al., 2012).

## Discovery of bioactive compounds from marine rare actinobacteria

*Actinobacteria* including *Streptomyces* contribute for approximately half of the characterized bioactive compounds up to date (Berdy, 2005). However, the chances of discovery of novel bioactive molecules from *Streptomyces* has significantly declined (Fenical et al., 1999), presumably due to easy chances of genetic exchange between species during evolution (Freel et al., 2011). Therefore, special attention is given to isolation, screening, and culturing of rare actinobacteria from rare environmental locations as marine sources. The list below summarizes some of the representative compounds isolated from diverse marine rare actinobacteria during last 10 years (Table 1A).

**Table 1:** Overview of achievements in study of bioactive molecules derived from marine rare actinobacteria.

**A. Examples of bioactive compounds isolated from various marine rare actinobacteria**				
**Compound name**	**Isolation source**	**Bacterial source**	**Biological activities**	**References**
**INDEPENDENT ISOLATES**				
Pseudonocardians	Deep-sea sediment of South China Sea	Pseudonocardia sp. SCSIO 01299	Antibacterial and cytotoxic	Li et al., 2011
Caerulomycins	Marine sediments from the seashore of Weihai, China	*Actinoalloteichus cyanogriseus* WH1-2216-6	Cytotoxic, antibacterial	Fu et al., 2011
Marinacarbolines,	Marine sediment sample from South China Sea	*Marinactinospora thermotolerans* SCSIO 00652	Antimalarial	Huang et al., 2011
Salinosporamides (Commercial name *Marizomib*)	Deep sea-water of Bahamas Islands, Bahamas	*Salinispora tropica* (strain CNB-392)	Cytotoxic	Feling et al., 2003; Williams et al., 2005
Abyssomicins	Sediment sample from the Sea of Japan, Japan	*Verrucosispora* sp. AB-18-032	Antibacterial	Bister et al., 2004; Riedlinger et al., 2004
Marinomycins	Sediment sample offshore of La Jolla, USA	*Marinispora* strain CNQ-140	Cytotoxic	Kwon et al., 2006
Levantilides	Deep-sea sediment Eastern Mediterranean Sea	*Micromonospora* M71-A77	Cytotoxic	Gärtner et al., 2011
Salinoquinones	Deep sea-water of Bahamas Islands, Bahamas	*Salinispora arenicola* CNS-325.	Cytotoxic	Murphy et al., 2010
Neomaclafungin	Marine sediment from Usa bay, Kochi Prefecture, Japan.	*Actinoalloteichus* sp. NPS702	Antifungal	Sato et al., 2012
Marthiapeptide A	Deep-sea sediment of the South China Sea	*Marinactinospora thermotolerans SCSIO 00652*	Antibacterial, Cytotoxic	Zhou et al., 2012
Lucentamycins	Sediment sample from Bahamas island, Bahamas	*Nocardiopsis lucentensis (strain CNR-712)*	Cytotoxic	Cho et al., 2007
Juvenimicin C	Sediment collected off the coast of Palau, USA	*Micromonospora* sp (CNJ-878)	Cancer chemo preventive	Carlson et al., 2013
Levantilide C	Shallow coastal waters near the island of Chiloe, Chile.	*Micromonospora* strain FIM07-0019	Antiproliferative	Fei et al., 2013
Nocapyrones	Sediment sample, Ulleung Basin, Eastern sea, Korea	*Nocardiopsis* sp.	Reduced the pro-inflammatory factor	Kim et al., 2013
Nocardiamides	Sediment sample from La Jolla Canyon, San Diego, California, USA.	*Nocardiopsis* sp. CNX037	Low antibacterial activity	Wu Z. C. et al., 2013
Cyanogramides	Marine sediments from the seashore of Weihai, China	*Actinoalloteichus cyanogriseus* WH1-2216-6	Multidrug-resistance (MDR) reversing activity	Fu et al., 2014
Taromycin	Marine sediment sample from La Jolla Submarine Canyon, San Diego, California, USA.	*Saccharomonospora* sp. CNQ-490	Antibacterial	Yamanaka et al., 2014
Lodopyridone	Marine sediment sample from La Jolla Submarine Canyon, San Diego, California, USA.	*Saccharomonospora* CNQ490	Modest cytotoxic activity	Maloney et al., 2009
Lynamicins	Marine sediment off the coast of San Diego, California, USA	*Marinispora* NPS12745	Antibacterial	McArthur et al., 2008
Saccharothrixones	Sediment sample from Heishijiao Bay, Dalian, China	*Saccharothrix sp. 10-10*	Cytotoxic	Gan et al., 2015
Saliniketals	Sediment sample from Island of Guam, USA	*Salinispora arenicola CNR-005*	Prevention of carcinogenesis	Williams et al., 2007a
Arenicolides	Sediment sample from Island of Guam, USA	*Salinispora arenicola* CNR-005	Moderate cytotoxicity	Williams et al., 2007b
Lagumycin B, Dehydrorabelomycin, Phenanthroviridone, WS-5995 A	Sediment sample from Cát Bà Peninsula, East Sea Vietnam	*Micromonospora* sp.	Cytotoxic	Mullowney et al., 2015
Dermacozines, Phenazine derivatives	Sediment sample from Mariana Trench	*Dermacoccus abyssi sp. nov*., strains MT1.1 and MT1.2	Cytotoxic and anti-oxidant	Abdel-Mageed et al., 2010
Fijiolides	Sediment sample from the Beqa Lagoon, Fiji	*Nocardiopsis* CNS-653	Inhibitor of TNF-α-induced NFκB activation	Nam et al., 2010
Fluostatin	Sediment sample from South China Sea	*Micromonospora rosaria* SCSIO N160	Antimicrobial	Zhang et al., 2012
Retimycin	Deep sea-water of Bahamas Islands, Bahamas	*S. arenicola* strain CNT-005.	Cytotoxic	Duncan et al., 2015
Sioxanthin	Deep sea-water of Bahamas Islands, Bahamas	*Salinispora tropica* CNB-440	Siderophore	Richter et al., 2015
Lobosamides	Sediment sample from Point Lobos, Monterey Bay, California, USA.	*Micromonospora sp*. RL09-050-HVF-A	Antitryposomal	Schulze et al., 2015a
Salinipostins	Sediment sample from Keawekaheka Bay, Hawai, USA	*Salinispora* sp. RL08-036-SPS-B	Antimalarial	Schulze et al., 2015b
Isomethoxyneihumicin	Sediment sample at Chichijima, Ogasawara, Japan	*Nocardiopsis alba* KM6-1	Cytotoxic	Fukuda et al., 2016
Nocarimidazoles	Sediment sample off the coast of southern California, USA	*Nocardiopsis* sp. CNQ115	Weak antibacterila	Leutou et al., 2015
Cyclomarine Cyclomarazine	Marine sediment from Palau, Republic of Palau	*S. arenicola* CNS-205	Anti-inflammatory	Schultz et al., 2008
**ISOLATES IN SYMBIOTIC ASSOCIATION**				
JBIR-65	Symbiont to unidentified marine sponge from Ishigaki Island, Okinawa Prefecture, Japan	*Actinomadura* sp. SpB081030SC-15	Anti-oxidant	Takagi et al., 2010
Nocapyrones	Symbiont to sponge *Halichondria panacea* from Baltic Sea, Germany	*Nocardiopsis* sp. HB383	Weak cytotoxic	Schneemann et al., 2010
Arenjimycin	Symbiont to ascidian *Ecteinascidia*	*Salinispora arenicola*	Antimicrobial and ytotoxic	Asolkar et al., 2010
	*Turbinate* from Sweetings Cay, Grand Bahama Island, USA			
Bendigoles	Symbiont to sponge *Suberites japonicas* from unspecified source	*Alctinomadura* sp. SBMs009	Antimicrobial and cytotoxic	Simmons et al., 2011
Thiocoraline	Symbiont to sponge *Chondrilla caribensis* from Florida Keys, USA	*Verrucosispora* sp.	Cytotoxic	Wyche et al., 2011
Peptidolipins	Symbiont to ascidian *Trididemnum orbiculatum* from Florida Keys, USA	*Nocardia* sp.	Antibacterial	Wyche et al., 2012
Anthracyclinones	Symbiont to tunicate *Eudistoma vannamei* from Taìba Beach, Ceará, Brazil	*Micromonospora* sp.	Cytotoxic	Sousa et al., 2012
Halomadurone	Symbiont to ascidian *Ecteinascidia turbinata*, from Florida Keys, USA	*Actinomadura* sp.	Active against neurodegenerative diseases	Wyche et al., 2013
Solwaric acids	Symbiont to ascidian, *Trididemnum orbiculatum* from Florida Keys, USA	*Solwaraspora* sp.	Antibacterial	Ellis et al., 2014
Forazoline A	Symbiont to ascidian, *Ecteinascidia turbinate from* Florida Keys	*Actinomadura* sp. WMMB-499	Antifungal	Wyche et al., 2014
Rifamycins	Symbiont to sponge, *Pseudoceratina clavata*. From Great Barrier Reef, Australia	*Salinispora* sp. strain M403	Antibacterial	Kim et al., 2006
Saccharothrixmicines	Symbiont to marine mollusk *Anadara broughtoni* from Sea of Japan	*Saccharothrix espanaensis* An 113	Antibacterial, Antifungal	Kalinovskaya et al., 2010
**B. Approaches used for production and structural/functional diversification of bioactive compounds derived from marine rare actinobacteria**				
**Compound name**	**Genus**	**Particulars**	**Biological activity**	**References**
Retimycin	*Salinospora*	MS/MS spectrum pattern based genome mining	Cytotoxic, Antibacterial	Duncan et al., 2015
Thiolactomycin	*Salinospora*	Antibiotic resistance gene based genome mining, heterologous expression	Bacterial fatty acid synthase inhibitor	Tang et al., 2015
Lomaiviticin	*Salinospora*	Bioactivity guided genome mining	Cytotoxic	Kersten et al., 2013
Salinosporamide K	*Salinospora*	Genome mining, metabolomics and transcriptomics	Cytotoxic	Eustáquio et al., 2011
Taromycin	*Saccharomonospora*	BCG Genome mining, heterologous expression	Antibacterial	Yamanaka et al., 2014
Enterocin	*Salinispora*	BCG Genome mining, heterologous expression	Antibacterial	Bonet et al., 2014
Fluostatins	*Micromonospora*	Heterologous expression	Antibacterial	Yang et al., 2015
Thiocoraline	*Micromonospora*	Heterologous expression	Cytotoxic	Lombó et al., 2006
Bromosalinosporamide	*Salinospora*	Precursor directed biosynthesis	Cytotoxic	Lam et al., 2007
Salinosporamide A	*Salinospora*	Precursor pathway modulation	Cytotoxic	Lechner et al., 2011
Salinosporamide X1, Salinosporamide X2	*Salinospora*	Combinatorial biosynthesis	Cytotoxic	McGlinchey et al., 2008
Salinosporamide X3	*Salinospora*	Mutasynthesis	Cytotoxic	Nett et al., 2009
Salinosporamide X4				
Salinosporamide X5				
Salinosporamide X6				
Salinosporamide X7				
Fluorosalinosporamide	*Salinospora*	Mutasynthesis	Cytotoxic	Eustáquio and Moore, 2008
Salinosporamides analogs	*Salinospora*	Chemobiosynthesis	Cytotoxic	Liu et al., 2009
Salinosporamide A	*Salinospora*	Total chemical synthesis	Cytotoxic	Reddy et al., 2004; Endo and Danishefsky, 2005; Kaiya et al., 2011; Logan et al., 2014
Homosalinosporamide	*Salinospora*	Total chemical synthesis	Cytotoxic	Nguyen et al., 2010
Salinosporamides analogs	*Salinospora*	Chemobiosynthesis	Cytotoxic	Liu et al., 2009
Salinosporamide E	*Salinospora*	Semi-synthesis	Cytotoxic	Macherla et al., 2005
Bromosalinospramide				
Iodosalinosporamide, Azidosalinosporamide, Hydroxysalinosporamide				
Methylsalinosporamide	*Salinospora*	Semi-synthesis	Cytotoxic	Manam et al., 2008
Tosylsalinosporamide				
Dansylsalinosporamide				
Hydroxysalinosporamide				
Flurosalinosporamide				

## Reinvigorating natural product discovery from marine rare actinobacteria

Though isolation and cultivation of marine rare actinobacteria is difficult, the development of novel and facile bacterial cultivation platforms such as hollow-fiber membrane chamber (HFMC) and iChip for *in situ* cultivation of previously unculturable microbial species have expanded the scope of natural product discovery (Aoi et al., 2009; Nichols et al., 2010). By utilizing rationally designed iChip platform, Ling et al. (2015) has successfully isolated previously uncultivable soil bacteria *Eleftheria terrae* and characterized its bioactive molecule (Ling et al., 2015).

**Figure 1:**
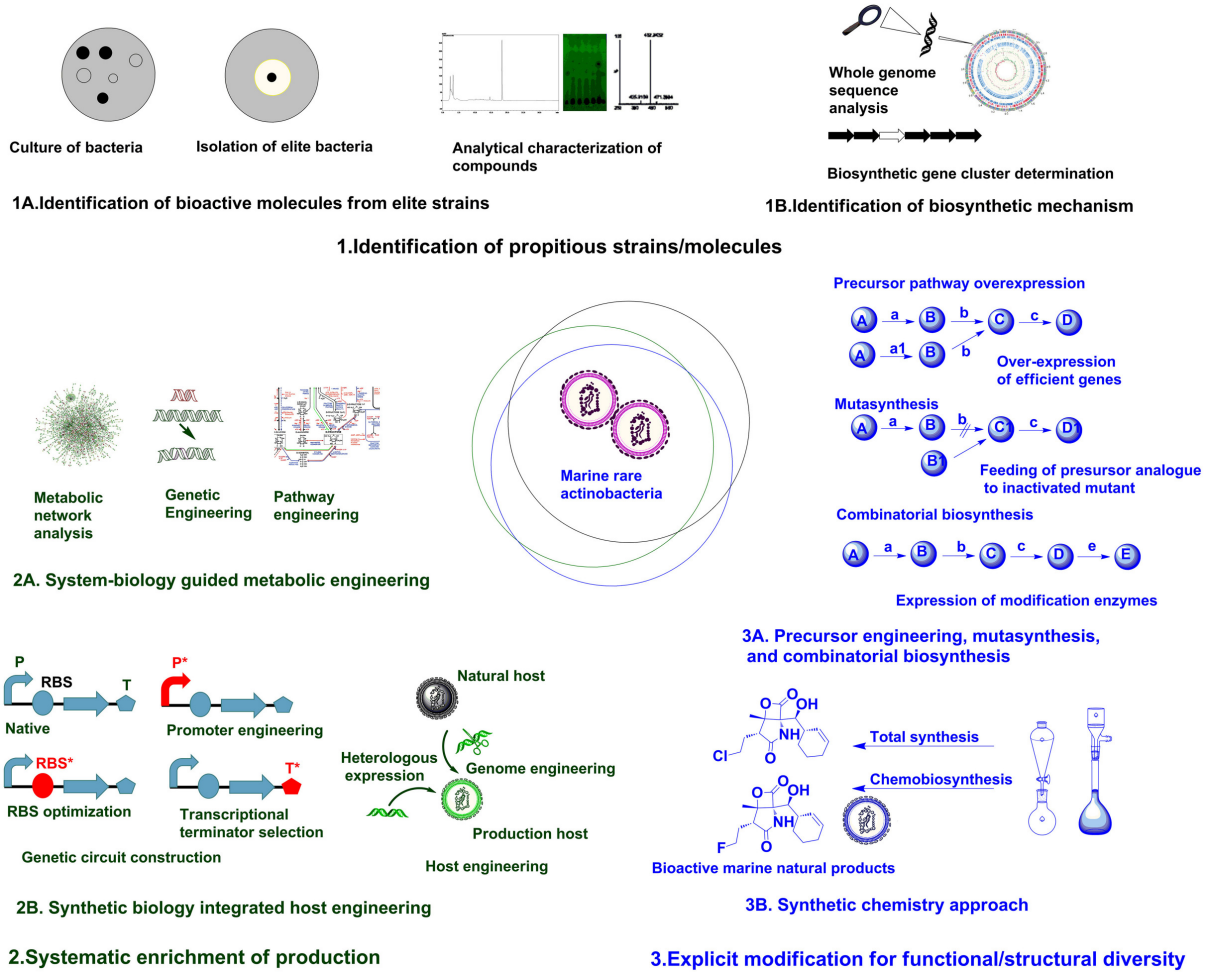
Different approaches for enhancing natural product discovery from marine rare actinobacteria.

It is assumed that strain divergence (phylogenetic or ecological) can have great impact on metabolism and biosynthetic pathway and result in novel chemistry and bioactivities, so research is focused on previously unexplored strains (Monciardini et al., 2014). However, it is unrealistic to assume that every unexplored strain can provide bioactive compounds (Donadio et al., 2010). Hence, systematic approaches need to be employed for utilizing the true potential of natural products from marine rare actinobacteria. Some of the key foundations can be categorized as:

1. Identification of target strains/molecules,
2. Systematic enrichment of production,
3. Explicit modification for functional/structural diversity.

1. Identification of target strains/molecules
  The accessible diversity of useful microbial molecules have almost been exhausted by traditional approaches, hence it is speculated that unstudied marine rare actinobacteria can provide reservoir of new microbial molecules (Schorn et al., 2016). Recently, direct connection of genomic information to biomolecule can be attained in culture independent approach as introducing environment (eDNA) into a suitable expression host (metagenomic libraries) (Handelsman, 2004). But, compound rediscovery due to similar strain replications is a major limitation of this approach. To maximize the capacity to mine metagenomes for attaining biomolecules with novel activities, there is requisite for parallel developments in techniques for bioactivity screening, isolation and separation methods, and analytical chemistry (Trindade et al., 2015). Robust techniques for analytical characterization of compounds (Figure 1A) based on UV absorbance, high pressure liquid chromatography (HPLC), mass spectrometry, and nuclear magnetic resonance (NMR) analysis can be used to scrutinize the discovery of new compounds (Liu et al., 2012). The techniques utilizing coupling of biochemical analytical methods with genome information such as, in glycogenomics (Kersten et al., 2013), peptidogenomics (Medema et al., 2014), and metabolomics (Maansson et al., 2016) are recent advances facilitating easy access to diverse biomolecules. The results of such analytical analysis can be subsequently compared against databases repositories, such as MarinLit, ChemSpider, Pubchem, etc., to avoid already known compounds (Forner et al., 2013). Hence, robust analytical facilities and comparison with reference databases can assist on characterization of diverse chemical structures.
  The prime focus in drug discovery is identification of new bioactive chemical or discovery of previously unreported biological activity with known chemical structure. High throughput screening (HTS) can provide easy means for evaluating desired bioactivities against an array natural products (Monciardini et al., 2014). The robust screening strategies ranging from the classic whole cell assays to more sophisticated antisense based assay have been reviewed elsewhere (Silver and Bostian, 1990; Singh et al., 2011; Farha and Brown, 2016). Recently, the integrative approach of metabolite profiling, bioactivity studies and taxonomic studies have been utilized for characterizing different marine actinobacteria and biological properties of metabolites produced by them (Betancur et al., 2017). Such integrative approaches can be fascinating tool for directly assessing bioactivities at preliminary stages of study.
  The next focus in drug discovery is understanding the biogenesis of bioactive molecule in producer strains. The rapid development of genome sequencing methods has revolutionized such studies by unveiling information about the whole genome architecture (Figure 1B). The challenge now is mining the data and connect the predicted biosynthetic gene clusters (BGC) to bioactive molecules. A plethora of *in silico* tools are available for determining the nature of gene clusters (Weber and Kim, 2016). The classic genome mining approach (focusing on unique biosynthetic enzyme) has transitioned to the concept of comparative genome mining (complete BGC to next BGC comparison) and culture independent-metagenome mining (Ziemert et al., 2016). Due to its efficacy in studying BGCs, the genome mining concept has been expanded to different marine rare actinobacteria for getting insight on biosynthesis mechanisms of different secondary metabolites. The analysis of genome sequence of *Micromonospora* sp. RV43, *Rubrobacter* sp. RV113, and *Nocardiopsis* sp. RV163 isolated from Mediterranean sponges revealed presence of numerous gene clusters of different secondary metabolites (Horn et al., 2015). The 5.2 Mb genome of marine rare actinobacteria, *Salinispora tropica* CNB-440 (Udwary et al., 2007) was interpreted using bioinformatics revealing at least 19 novel secondary metabolite BCGs. Later, diverse compounds have been characterized from *S. tropica*, including anticancer agent salinosporamide A, lymphocyte kinase inhibitor lymphostin, DNA-cleaving agent calicheamicin, novel lysin-primed polyene macrolactam polyketide, and various siderophores (Kersten et al., 2013). Biosynthetic analysis of the draft genome of *Saccharomonospora* sp. CNQ490 has revealed 19 conspicuous BGC, indicating diverse secondary metabolic capacity (Yamanaka et al., 2014). Using precise bioinformatics tools, 75 genomes from closely related *Salinospora* species were compared and 124 distinct prominent BCGs were predicted which are far greater than known compound classes from these bacteria (Ziemert et al., 2014). Duncan et al. (2015) has simultaneously compared a large number of complex microbial extract in a large number of *Salinispora* species. This molecular networking was coupled with genome sequence data for comparative analysis of metabolite profile and BCG to develop pattern-based genome mining (PBGM) approach. Concurrently, a novel non-ribosomal peptide, retimycin A was isolated and characterized based on genome and metabolome analysis (Duncan et al., 2015). Therefore, genome mining approach has provided new avenues on discoursing novel natural products from marine rare actinobacteria.
2. Systematic enrichment of production
  Generally, genome information is the starting point for pathway discovery. Various “omics” based tools have been employed for engineering pathways for secondary metabolite production in various actinobacteria (Chaudhary et al., 2013; Hwang et al., 2014). But the lack of full understanding of physiological transition stage for secondary metabolite production is a major consideration during manipulation of cellular processes using metabolic engineering (Licona-Cassani et al., 2015). Engineering primary metabolism for enhancing the pools of building blocks without compromising the growth is a major constraint in most metabolic engineering approaches (Olano et al., 2008). System biology protocols have been successfully used to study physiological parameters, leading to the discovery of the activation of NPs biosynthesis and manipulation of pathways (Licona-Cassani et al., 2015). Genome scale metabolic models are valuable for predicting organisms' phenotypes from genotypes basically by providing simulated mathematical prediction of cellular behavior under different genetic and physiological conditions (Henry et al., 2010; Ates et al., 2011). Community system biology approaches provide understanding about the complex relationship of individual members in a community and the modes of interactions they are engaged (Zengler and Palsson, 2012). The systematic application of systems biological approaches as metabolic network analysis coupled with pathway engineering or genetic engineering (Figure 2A) from a single strain to the larger community level can provide breakthrough in rational metabolic engineering approaches.
  Synthetic biology is particularly focused on precise design and construction of new biological systems (metabolic pathways or genetic circuits) that are not prevalent in nature (Andrianantoandro et al., 2006). Previously, efforts in synthetic biology have been largely focused on creating and perfecting genetic devices. But the current focus is directed to customizable larger scale system engineering by assembling devices or modular organizations (Purnick and Weiss, 2009). Most often, biologically valuable natural products are produced in lower titer or are cryptic under normal laboratory conditions, whereas many rare actinobacteria are not amenable to genetic manipulation. Hence, in such cases transferring natural products biosynthesis into well-developed heterologous host is a logical approach for producing parent NPs or generating novel analogs through biosynthetic engineering (Wenzel and Müller, 2005). Direct cloning and refactoring of previously silent lipopeptide gene cluster of *Saccharomonospora* sp. CNQ490 have been achieved by heterologous expression in *Streptomyces coelicolor* to yield taromycin A by Transformation Assisted Recombination (TAR)-based genetic platform (Yamanaka et al., 2014). Besides, tuning of metabolic pathway by altering promoters (Siegl et al., 2013; Wang et al., 2013), terminators (Pulido and Jimenez, 1987), and RBS (Bai et al., 2015) and/or host manipulation by genome engineering (Siegl and Luzhetskyy, 2012; Tong et al., 2015) are providing new avenues for systemic level metabolic engineering of actinobacteria. Promoter exchange (Horbal et al., 2012) and the use of exogenous principal sigma factor (σHrdB) (Wang et al., 2014) have been utilized for increasing teicoplanin in an industrial strain of *Actinoplanes teichomyceticus*. Approach for constructing genetic circuit or holistic host engineering (Figure 2B) can be an effective approach for designing and synthesizing unnatural but effective molecules from marine rare actinobacteria.
3. Explicit modification for functional/structural diversity
  Fundamentally, engineering or modulating the precursor pathways can lead to enhancement or diversification of natural products (Dhakal et al., 2016). Combinatorial biosynthesis exploits the shuffling of anabolic pathways by precursor directed biosynthesis, enzyme level modulations, and pathway level recombination, leading to novel natural products (Sun et al., 2015; Winn et al., 2016). The precursor-directed in-situ synthesis (PDSS) has been successfully employed for generating new congeners of saccharothriolides from *Saccharothrix* sp. A1506 (Lu et al., 2016). Such type of precursor modulations can be manifested chemically or biologically to generate structural diversity in compounds from marine rare actinobacteria. Mutasynthesis is another variant of modulation of anabolic pathway by generating mutant strain deficient in key aspects of biosynthetic pathway and substituting natural precursor with analog of precursor to produce new natural products (Kennedy, 2008). Mutasynthesis couples the power of chemical synthesis with molecular biology to create diverse derivatives of medicinally valuable natural products (Weissman, 2007). One such example is the production of fluorinated analog fluorosalinosporamide. It has better proteasome inhibition and cytotoxic activity than naturally produced salinosporamides isolated from various *Salinispora* species (Feling et al., 2003). The halogenase gene *salL* in *Salinispora tropica* has been inactivated and 5′-fluoro-5′-deoxyadenosine, a fluorinated analog of its natural precursor 5′-chloro-5′-deoxyadenosine, has been used to generate fluorosalinosporamide by chemistry mediated mutasynthesis (Eustáquio and Moore, 2008). In another approach, *salL* was replaced by fluorinase gene *flA* from *Streptomyces catteleya*. The mutant strain *salL*^−^*flA*+ produced fluorosalinosporamide in the presence of inorganic fluoride (Eustáquio et al., 2010). Moreover, combinatorial biosynthetic approach by feeding L-3-cyclohex-2′-enylalanine (CHA) residue in SalX disruption mutant of *S. tropica* enabled the generation of other unnatural salinosporamide derivatives such as salinosporamide X1 and salinosporamide X2, with lower activity (McGlinchey et al., 2008). But in another approach utilizing mutasynthetic approach with fine-tuned feeding of readily available amino acid precursors to SalX disruption mutant of *S. tropica* led to generation of many salinosporamide derivatives. Among them salinosporamide X7 exhibited equal to slightly improved cytotoxic potential than the natural counterpart (Nett et al., 2009). Hence, such approaches of precursor engineering, mutasynthesis, and combinatorial biosynthesis (Figure 3A, Table 1B) can be rationally utilized to diversify structure and perform structure-activity relationship studies of versatile molecules from various marine rare actinobacteria.
  The advent of combinatorial synthetic chemistry has created huge excitement in the pharmaceutical industry by generating libraries of millions of compounds which could be screened by HTS (Butler, 2004). The total synthesis of complex natural products offers greater potential for direct access to bioactive molecule from marine sources. However, large scale production of complex natural product remains elusive due to low yields and high cost (Yeung and Paterson, 2005). Recent achievement as total synthesis of natural products in absence of protecting groups can lead to development of superior molecules with greater flexibility (Young and Baran, 2009). The generation of microbial chemicals by total enzymatic synthesis has been used as alternative to total chemical synthesis (Cheng et al., 2007). There have been ample of examples illustrating improvement in physical and biological properties of natural products (including many marine natural products) by chemical modifications, semisynthesis, mutasynthesis, and chemobiosynthesis (Hamann, 2003; Kennedy, 2008) mediated by biological and chemical techniques. Bioinspired total synthesis of salinosporamides and structurally related derivatives have provided access to novel functionalities of tremendously effective molecule (Nguyen et al., 2010; Chen et al., 2012). Suitable integration of synthetic chemistry (Figure 3B, Table 1B) with biological production system can be utilized for generating structurally and functionally diverse analogs/derivatives of target molecule. One of the successful example illustrating application of synthetic chemistry in marine natural products is rationalized for structural/functional diversification of salinosporamides (Baran et al., 2007; Potts and Lam, 2010). The synergy between genome sequencing, mass spectroscopy based analysis and bio-inspired synthesis have been utilized for studying biosynthetic mechanism and structural diversification of nocardioazine B from Nocardiopsis sp. CMB-M0232 (Alqahtani et al., 2015). Hence, it is no doubt that rational integration of biological processes and chemical techniques (Dhakal and Sohng, 2015, 2017) can provide new foundations for drug discoveries from marine rare actinobacteria.

## Future outlook

As evident from examples above, the innovative methods for procurement of bioactive molecules from potent strains, efficient production and/or modifications by biological and chemical methods can assist in harnessing the full potential of biomolecules derived from marine rare actinobacteria. Further, tuning of structural and functional properties based on structure activity relationship studies can lead to development of superior analogs. But the prime focus should be on application of cutting edge translational research, such as transferring the achievements of discovery or synthesis of such biomolecule to the industrial bench-tops and clinics. The successful collaboration between biologists/chemists in academics and/or pharmaceutical companies can open new avenues for development of highly effective drugs. Salinosporamide A (*Marizomib*) has been a significant representation of compound derived from marine rare actinobacteria leading to phase trials. It is no doubt that exploration of new candidate strains with sophisticated techniques will certainly unravel tremendous opportunities to identify novel natural products and improve their applicability by structural/functional diversifications.

## Author contributions

DD, ARP, BS, and JS made substantial, direct, and intellectual contribution to the work, and approved it for publication with full consent.

### Conflict of interest statement

The authors declare that the research was conducted in the absence of any commercial or financial relationships that could be construed as a potential conflict of interest.
